# U.S. climate change policy must include vector-borne disease prevention

**DOI:** 10.3389/fpubh.2026.1792352

**Published:** 2026-05-07

**Authors:** Sarah E. Dumas, Katherine R. O'Brien, Maya M. Rao, Timothy H. Holtz

**Affiliations:** Sumner M. Redstone Global Center for Prevention and Wellness, The George Washington University Milken Institute School of Public Health, Washington, DC, United States

**Keywords:** climate adaptation, climate change, disease surveillance, public health policy, vector-borne diseases (VBDs)

## Introduction

Climate change is altering vector-borne disease (VBD) risk in the United States. The relationship is complex; changes in land use, ecosystem degradation, urbanization, and shifts in human behavior also contribute to changing risk. However, there are clear, documented links between climate factors and VBD risk. The survival, reproduction, and activity of arthropod vectors are highly dependent on environmental conditions. There is strong evidence that changes in temperatures, precipitation, and humidity affect the reproduction, development, survival, and biting rate of vectors, most notably mosquitos and ticks (1). For example, higher average temperatures and increased precipitation promote mosquito breeding and survival (2). Increased extreme weather events, especially heavy rainfall and flooding, create abundant breeding habitats for mosquitos, leading to population surges (3), though the impact on VBDs is not well established.

These shifts have reshaped the landscape for VBD risk in the U.S. Warmer temperatures, in particular, have contributed to expanded or shifted suitable geographic ranges for both vectors and hosts (4–6). In endemic areas, longer summers and milder winters have lengthened vector breeding and transmission seasons (7). Transmission intensity has increased, in part due to climate-associated shifts in vectorial capacity (5).

These risks are not abstract; they are already impacting American's health. Once considered tropical or regional, VBDs like dengue, West Nile, Lyme disease, anaplasmosis, and babesiosis are becoming more widespread (8–12). The Centers for Disease Control and Prevention (CDC) reported 6,543 cases of locally acquired dengue in the U.S. in 2024, more than in the previous 10 years combined (9), and climate change is considered one of several contributing factors (13). Similarly, in the past 40 years, Lyme disease incidence has increased significantly in the U.S. (10). This increase is primarily driven by expanded geographic range of the *Ixodes* tick vectors (14), and evidence suggests that warming temperatures, alongside host abundance and land use shifts, may be facilitating this expansion (4, 15).

Despite this evidence, we argue that public health policies have left the American public vulnerable and unprepared. The 2020 *National Public Health Framework for the Prevention and Control of Vector-Borne Diseases in Humans* neglected the role of climate in shaping VBD risk in the country (16). Although a subsequent national strategy corrected this omission by acknowledging the need to understand climate impacts on vectors and pathogens (17), it remains inadequately operationalized. Many relevant government resources, including the de-funded U.S. Global Change Research Program's National Climate Assessments and the Climate and Health Assessment, are no longer available

on government websites, raising questions about sustained federal commitment. There is no national adaptation policy mandating climate-informed VBD planning across states and agencies.

Limited sustained federal action since 2024 has shifted the burden to state and local jurisdictions, creating capacity gaps and inconsistent surveillance. The CDC's Building Resilience Against Climate Effects (BRACE) framework has been adopted by a number of state and local health departments (18), and it explicitly frames VBDs as a key climate vulnerability (19). Unfortunately, while some state climate adaptation plans mention VBD risk, few have operational response plans. For example, in Colorado, the state with the greatest number of reported West Nile virus cases in 2025, the state's Climate Plan outlines efforts to monitor disease trends and explore “possible links to climate variables,” but it stops short of recommending any specific prevention or adaptation actions (20). Similarly, in Pennsylvania, which has consistently reported large numbers of Lyme disease cases over the past decade, the most recent Climate Action Plan mentions Lyme disease only once and recommends surveillance efforts “to better understand climate-related health impacts.” However, the plan fails to provide concrete actions for addressing these impacts (21).

## What Americans think

To explore Americans' risk perceptions and behaviors around climate change and VBDs, we fielded questions as part of a Verasight survey [online, November 14–20, 2025; report available online (22)]. Respondents (*n* = 3,000 across three independent modules, *n* = 1,000 per module) were sampled from Verasight's nationally representative panel of U.S. adults, and responses were weighted to population benchmarks (margin of error = ±3.2%). We independently analyzed the data; Verasight had no role in analysis or interpretation.

We found that over half of Americans (54%) are concerned that climate change will increase their risk of contracting an infectious disease (Table 1). Black and Hispanic Americans were more likely to express concern compared to White Americans (64% and 61% vs. 49%, *p* = 0.03 for both), reflecting both disproportionate climate impacts (23, 24) and structural inequities that influence differential exposure and health burden (25, 26). We also found that Americans under the age of 50 were more concerned about the risk of climate-related infectious diseases than older Americans (58% vs. 49%, *p* < 0.01). This aligns with previous research showing that young people are more worried about climate change and its impacts than older generations (27–29). This concern cuts across geography, with no meaningful regional or urban-rural differences, underscoring that climate-driven health risks are a national issue.

**Table 1:** Weighted distribution of attitudes and protective health behaviors between sociodemographic groups among U.S. adults (*n* = *1,000* per question).

	% Reporting (95% Confidence Limits)				
Sociodemographic group	Concerned that climate change could increase risk of disease^1^	Used mosquito repellant when going outside^2^	Stayed inside when mosquitos usually bite^3^	Checked for ticks after risk exposure^4^	Support integrating VBD prevention into national climate policies^5^
**Overall**	**54% (50.6, 57.1)**	**32% (29.5, 35.5)**	**30% (27.2, 33.2)**	**28% (25.5, 31.2)**	**81% (78.4, 83.5)**
Age					
Under 50 (ref; *n* = 477–488)^†^	58% (53.9, 63.1)	35% (31.0, 39.7)	33% (28.4, 37.1)	29% (25.1, 33.4)	84% (80.8, 87.6)
50 and over (*n* = 512–523)	49%^**^ (44.1, 52.9)	29%^*^ (25.2, 33.3)	27% (23.3, 31.2)	27% (23.3, 31.2)	77%^**^ (73.4, 81.0)
Gender					
Male (ref; *n* = 433–471)	50% (45.5, 55.3)	27% (22.9, 31.3)	25% (21.3, 29.6)	27% (22.4, 30.7)	79% (75.4, 83.1)
Female (*n* = 523–561)	57% (52.9, 61.4)	37%^*^ (33.0, 41.5)	34%^**^ (30.1, 38.6)	30% (25.8, 33.8)	82% (79.0, 85.7)
Party identification					
Democrat (ref; *n* = 291–325)	74% (69.2, 79.3)	35% (29.5, 40.7)	35% (29.1, 40.5)	24% (19.3, 29.4)	91% (87.8, 94.4)
Independent (*n* = 272–298)	52%^**^ (46.2, 58.4)	34% (28.3, 39.5)	32% (26.1, 37.2)	33% (27.9, 38.9)	81%^**^ (76.5, 86.2)
Republican (*n* = 297–333)	34%^**^ (28.4, 39.8)	28% (23.3, 33.4)	25%^*^ (20.5, 30.1)	27% (22.4, 32.2)	71%^**^ (66.0, 76.5)
Race and ethnicity					
White (ref; *n* = 635–656)	49% (45.0, 52.9)	33% (29.1, 36.7)	29% (25.0, 32.2)	34% (29.8, 37.3)	78% (75.0, 81.7)
Black (*n* = 93–105)	64%^*‡^ (53.8, 74.9)	27% (18.7, 36.2)	35% (25.4, 45.1)	23% (14.5, 31.5)	78% (69.6, 86.9)
Hispanic (*n* = 153–179)	61%^*^ (52.8, 69.2)	33% (26.1, 40.3)	30% (23.0, 36.9)	17%^**^ (11.6, 22.9)	87% (81.2, 91.6)
Another race and/or ethnicity (*n* = 80–98)	57%^‡^ (46.9, 67.5)	35%^‡^ (24.3, 46.1)	35%^‡^ (23.8, 45.7)	24%^‡^ (14.4, 33.8)	89% (82.7, 96.0)
Region					
Northeast (ref; *n* = 186–204)	54% (47.0, 61.2)	32% (24.6, 38.5)	23% (16.6, 29.1)	31% (23.9, 37.6)	88% (83.8, 93.0)
Midwest (*n* = 192–211)	49% (42.0, 56.1)	29% (22.7, 36.0)	28% (21.0, 34.3)	31% (24.4, 37.6)	79%^*^ (73.3, 85.0)
South (*n* = 343–381)	53% (47.8, 58.9)	37% (31.8, 41.8)	37%^**^ (31.5, 41.6)	32% (27.0, 36.7)	81% (76.2, 84.9)
West (*n* = 228–346)	59% (52.3, 65.5)	30% (23.7, 36.0)	27% (21.3, 33.2)	16%^**^ (11.3, 21.2)	78%^*^ (72.1, 83.0)
Metropolitan area					
Metropolitan (ref; *n* = 861–880)	54% (51.0, 57.9)	32% (29.0, 35.5)	29% (26.2, 32.5)	26% (23.0, 29.0)	81% (78.3, 83.7)
Non-metropolitan (*n* = 109–126)	50% (41.6, 59.0)	36% (27.3, 44.8)	35% (26.7, 44.0)	41%^**^ (31.6, 49.4)	80% (72.2, 88.1)

^*^p < 0.05, ^**^p < 0.01;

^1^How concerned are you that climate change could increase your own risk of getting an infectious disease, like Lyme disease or West Nile virus? (% responding “somewhat concerned” or “very concerned”); ^2^In the past year, which of the following have you done to protect your family's health from the effects of climate change? (% selecting, “Used mosquito repellent when going outside”); ^3^(% selecting, “Stayed inside when mosquitoes usually bite more”); ^4^(% selecting, “Checked for ticks after walking in the woods or forest”); ^5^Would you support or oppose including measures to prevent mosquito- and tick-borne diseases (such as Lyme disease or West Nile Virus) as part of national climate change policies? (“% selecting, “Strongly support” or “Somewhat support”).

“Ref” is the reference category. ^†^Unweighted subgroup sample sizes vary by survey module. ^‡^Potentially unreliable estimate based on a coefficient of variation >20% or margin of error >10 percentage points. Interpret with caution.

## Americans are not protecting themselves

Despite their concern, Americans' protective behaviors do not necessarily align with their perceived risk. Only about one in three Americans reported using mosquito repellent before going outside in the past year (32%). Older adults were especially unlikely to use mosquito repellent (29% vs. 35%, *p* = 0.04), as were men (27% vs. 37%, *p* = 0.02). A similar proportion of Americans reported staying inside when mosquitoes are most active (30%), with men and Republicans being least likely to do so (25% and 25%, respectively). Only about one in four Americans checked themselves or their children for ticks after a risk exposure (28%); this was especially uncommon in the West (16%). While low protective behavior may in part reflect individuals' own assessments of their personal VBD risk right now, the gap between perceived future risk and protective behaviors may be lessened through clear state and local government guidance, targeted public health messaging, and multisectoral plans that integrate VBD risk and climate change.

## There is strong public support for integrating VBD prevention into climate policy

Indeed, four out of five Americans support integrating VBD prevention measures into national climate policies (81%). Despite small differences, support was strong across age, gender, race and ethnicity, region, and urbanicity. Even among the 21% of respondents who said they are not concerned about the health effects of climate change, over half (55%) support including measures to prevent mosquito- and tick-borne diseases in national climate change policies. While polls of policy proposals routinely attract high agreement, these findings nonetheless suggest public openness to change and readiness for government leaders to take action.

Some limitations to these data should be noted. First, the response rate (8.3%)—while similar to other high-quality population-base surveys in the U.S. (30, 31)—is low. Survey weights correct for differential response patterns and reduce the risk of nonresponse bias. Second, perceptual survey data can be biased. For instance, concern may not reflect actual risk, self-reported behaviors are subject to social desirability bias, and question framing may have inflated support for integrated VBD/climate policy. Finally, the data are cross-sectional and do not capture changes in risk perception or behaviors over time.

## Recommendations

Based on these findings, we propose five recommendations for advancing climate-informed VBD prevention in the U.S. Because climate interacts with land use, vector ecology, and human behavior, effective interventions will integrate climate-informed actions with established public health and environmental approaches. First, we recommend that VBD prevention and control should be integrated into state and local climate adaptation plans. There is strong public support for VBD planning, but limited government action to date. Recent shifts in federal priorities related to climate change and public health increase the importance of state and local leadership. Wisconsin's Climate and Health Adaptation Plan is a successful example, which prioritizes VBDs through surveillance, awareness messaging, remediation, and collaboration with health providers (32).

Second, we propose that expanded surveillance systems for vectors and VBD pathogens should be integrated with climate-informed early warning systems (CIEWS). Routine active surveillance monitors trends in vector abundance, distribution, and pathogen presence over time, informing timely, targeted interventions that can mitigate VBD transmission. Surveillance also provides baseline data for evaluating the impact of control efforts. The National Association of County and City Health Officials provides resources for local health departments, including a vector control “menu,” a worksheet for estimating costs, and guides for conducting assessments and evaluating interventions (33). By incorporating climate and environmental variables, CIEWS can more accurately predict transmission risks and inform outbreak prevention. Although a substantial body of literature has established the conceptual and methodological foundations for CIEWS globally (34), challenges remain in translating predictive models into operational public health tools. These efforts would also benefit from federal coordination to standardize vector surveillance across states, address local capacity gaps, and harmonize interoperable data systems.

Third, we suggest scaling up community-level education, particularly in historically marginalized communities that face disproportionate climate-related health risks. Educational efforts should emphasize actionable steps and ensure access to necessary resources, including free or low-cost repellents. The CDC provides free communication resources as part of their “Fight the Bite” campaign, which can be adapted for local contexts (35). Targeted messaging to those least likely to follow recommended protective practices could be delivered through collaboration with physicians or other trusted messengers. Partnerships with worker protection and occupational safety agencies can help address VBD risks among outdoor workers who face elevated exposures. Posted bulletins in areas with high tick activity can inform the public about the risks of TBDs and how to avoid ticks, perform tick checks, and safely remove attached ticks.

Fourth, we believe that sustained funding and dedicated resources for local programs are essential to support surveillance, vector control, and community education. In practice, this will require multi-year, predictable funding streams that allow local agencies to maintain staffing, training, and supplies rather than relying on short-term emergency allocations. States and cities could establish dedicated line items for critical vector control activities, while federal support could be expanded through grant mechanisms modeled on the CDC's Climate-Ready States and Cities Initiative. This would strengthen workforce capacity, enable long-term planning, and support climate-resilient infrastructure with co-benefits for vector control, such as green infrastructure designs that reduce standing water in urban environments (36). Structural investments should prioritize under-resourced neighborhoods that would most benefit from vector control activities and improvements in drainage infrastructure.

Finally, we suggest that investment in research and innovation for vector control is essential to advance safe, effective, and environmentally responsible prevention strategies. Vector control interventions must be rigorously evaluated to ensure they do not pose unintended risks to human health, non- target species, or the environment. Predictive modeling can be used to assess the effectiveness and potential downstream impacts of prevention and control strategies under changing climate conditions. Novel approaches, such as sterile insect technique (SIT) programs and *Wolbachia* mosquito infection, offer targeted, non-chemical control strategies that should be piloted and scaled up (37). These research efforts should be grounded in a One Health approach that supports cross-sectoral collaboration across human health, veterinary medicine, wildlife and environmental agencies, agricultural sectors, climate sciences, and emergency management partners. Practical mechanisms could include joint surveillance platforms that integrate human, animal, and environmental data; cross-sector advisory groups to guide local and state response planning; and communication channels to facilitate routine coordination. This cross-sector collaboration will help ensure that vector control strategies are effective, ecologically sound, and responsive to emerging climate-related risks.

## Conclusion

Our survey results show that Americans recognize the threat of climate-driven infectious diseases and broadly support solutions. Because prevention and early intervention are substantially more cost-effective than outbreak response, integrating climate-informed public health strategies represents both a public health and economic imperative. However, action remains limited across levels, from individual behavior change to policy implementation at the local, state, and national levels. To close the gap, two initial steps are immediately feasible: incorporating VBD considerations into upcoming state climate adaptation plan revisions and strengthening vector surveillance systems to account for climate-sensitive shifts in risk. These actions will guide more timely detection, targeted control efforts, and consistent planning across jurisdictions. As climate change accelerates amid shifting federal priorities, state and local leaders will play an increasingly critical role in filling gaps in VBD preparedness and prevention.
